# Personality Traits and Coping Strategies Relevant to Posttraumatic Growth in Patients with Cancer and Survivors: A Systematic Literature Review

**DOI:** 10.3390/curroncol29120754

**Published:** 2022-12-06

**Authors:** Klara Knauer, Anne Bach, Norbert Schäffeler, Andreas Stengel, Johanna Graf

**Affiliations:** 1 Psychosomatic Medicine and Psychotherapy, University Hospital Tübingen, 72076 Tübingen, Germany; 2 Comprehensive Cancer Center Tübingen-Stuttgart, Section Psychooncology, University Hospital Tübingen, 72070 Tübingen, Germany; 3 Charité Center for Internal Medicine and Dermatology, Department for Psychosomatic Medicine, Charité-Universitätsmedizin Berlin, Corporate Member of Freie Universität Berlin, Humboldt-Universität zu Berlin and Berlin Institute of Health, 10117 Berlin, Germany

**Keywords:** cancer, survivor, posttraumatic growth, PTG, Post-traumatic Growth Inventory, illness coping, personality, psycho-oncology

## Abstract

The possibility of positive psychological changes after cancer, namely, posttraumatic growth, is a growing field of research. Identifying personality traits and coping strategies related to posttraumatic growth may help find vulnerable individuals as well as promote helpful coping strategies to help more patients make positive changes at an early stage. The aim of this systematic literature review is to provide an overview of the quantitative data on coping strategies and personality traits associated with posttraumatic growth in patients with cancer and cancer survivors as well as the methods used in included studies. A systematic literature search was conducted using five databases (PubMed, PubPsych, PsycInfo, Web of Science, and PSYNDEXplus). The 70 reports of included studies assessed posttraumatic growth using questionnaires in a sample of patients with cancer or survivors. In addition, associations with a personality trait or coping strategy had to be examined cross-sectionally or longitudinally. All 1698 articles were screened for titles and abstracts by two authors, after which disputed articles were reviewed by a third author. Afterwards, articles were screened for full texts. Most studies had a cross-sectional design and used a sample of patients with breast cancer. Coping strategies have been researched more than personality factors. The personality traits of resilience, hardiness, dispositional positive affectivity, and dispositional gratitude seem to be related to posttraumatic growth, while the Big Five personality traits (openness to experience, conscientiousness, extraversion, agreeableness, neuroticism) have been less researched and/or seem to be unrelated. The use of social support, religious coping, positive reframing, and reflection during illness as coping strategies seems to be related to posttraumatic growth. The findings can be used for the development of interventions. Future studies should investigate associations longitudinally.

## 1. Introduction

Receiving a diagnosis and undergoing treatment for cancer are potentially traumatic events with negative psychological impacts, such as causing distress and symptoms of posttraumatic stress disorder (PTSD) [1,2,3]. Simultaneously, the struggle with such highly challenging life crises can have a positive impact, which is often referred to as posttraumatic growth (PTG) [4,5,6]. It can be influenced by factors such as personality traits and coping strategies. Our aim was to summarize studies that investigated relationships of personality traits and/or coping strategies with PTG. This can be of use for the development of interventions.

The experience of cancer can entail a series of traumatic events. Cancer can be regarded as a chronic stressor that can produce similar traumatic reactions as an acute traumatic event [7]. Half of patients with cancer have high levels of distress [2]. A high proportion of patients with cancer experiences symptoms of PTSD, and the probability of PTSD in cancer survivors is higher than that in the general population [3,8]. On the other hand, patients with cancer and survivors also report positive psychological changes [9,10]. The concept of posttraumatic growth was originally used for trauma victims but has long been extended to other serious illnesses such as cancer [11]. It entails five domains extracted through factor analysis of qualitative data of people who experienced stressful events or crises: greater appreciation of life and changed sense of priorities; warmer, more intimate relationships with others; a greater sense of personal strength; recognition of new possibilities or paths for one’s life; and spiritual development. It can be measured with the validated Posttraumatic Growth Inventory (PTGI) and is understood as a process that develops over time [6]. One fifth of long-term cancer survivors report moderate to high PTG, which is higher than that in the general population [10,12]. Still, the reported PTG of cancer survivors is lower than that of people working in a specific profession such as firefighters, veterans, and intensive care staff and people experiencing a series of adverse life events in general [11,13]. Existing research on the topic has shown that PTG has a practical impact as it is related to distress and PTSD symptoms [14]. For example, there are indications that the absence of PTG is a risk factor for later depression [15]. It is suggested that a certain amount of distress is necessary for development of PTG and that the development of PTG is associated with less distress later in life [16,17,18].

Personality differences predict the experience of benefits from adverse events [19]. In the original model of growth by Tedeschi and Calhoun [5,6], personality characteristics are described as playing a key role in the development of PTG. Personality traits are “relatively stable, consistent, and enduring internal characteristic that [are] inferred from a pattern of behaviors, attitudes, feelings, and habits in the individual” [20]. They found extraversion, openness to experience, and optimism to be related to PTG and suggested that certain personality types tend to cope with negative events in ways that lead to growth. Important personality traits such as the Big Five [21] have not yet been included in systematic reviews of PTG in cancer.

Apart from personality traits, the model of growth [5,6] describes coping strategies as playing a key role in the development of PTG. Coping strategies are mechanisms people use to deal with demands that exceed their own resources. They can entail behavior as well as cognitive processes [22]. Coping can be assessed situationally as well as dispositionally. There are different ways of categorizing coping strategies, for example, into problem-focused, emotion-focused, and less useful strategies [23]; however, no consensus exists. Tedeschi and Calhoun emphasized the role of certain cognitive processes and social support for PTG. These variables were later included in models explaining the development of PTG and reviewed systematically in people who experienced traumatic events in general [24,25,26]. Certain coping strategies play a crucial role in the development of PTG in certain cancer types such as breast cancer and oral cavity cancer. Systematic reviews found associations of PTG with the use of social support, religious, problem-focused and active coping, positive reinterpretation, acceptance, and humor [27,28]. It is unclear if these relationships can be generalized to all cancer types.

For the development of specific interventions enhancing PTG, it is necessary to understand the influential factors. PTG can be enhanced in patients with cancer and survivors through interventions, as shown in a meta-analysis of randomized-controlled trials [29]. Such interventions are most effective when mindfulness-based methods, including e-health, are used, when they are applied during acute cancer treatment and when participants have a breast cancer diagnosis and not another cancer diagnosis [29,30]. A short-term promotion of PTG might mitigate negative distress, and a long-term one might increase well-being [18]. The interventions included in meta-analyses are broad and not designed with a primary focus on PTG [31]. It is recommended to develop “systematic and focused” intervention strategies “focusing on the key process … and influential factors in the process of PTG” [29]. One suggested new intervention approach is to promote coping strategies in order to enhance PTG [27]. To identify vulnerable patients, it can be helpful to know what distinguishes patients with cancer and survivors who report high PTG from those who report low PTG in terms of their personality.

There is therefore a need for more research about the underlying processes. In conclusion, identifying relevant personality traits and coping strategies related to PTG may help identify vulnerable individuals early on, as well as promote helpful coping strategies to support more patients in making positive changes. The aim of this systematic literature review was therefore twofold: (1) to identify methods that have been used in studies to investigate the relationship between coping strategies and PTG; (2) to identify associations that have been found between different personality traits and coping strategies and PTG.

## 2. Materials and Methods

The systematic review was conducted according to the Preferred Reporting Items for Systematic Reviews and Meta-Analyses (PRISMA) statement criteria [32]. The review protocol was registered in advance in PROSPERO, the International Prospective Register of Systematic Reviews (CRD42022304224).

### 2.1. Search Strategy

We searched five databases, PubMed, PubPsych, PsycInfo, Web of Science, and PSYNDEXplus on 10 September 2022. The following combination of search terms was used: (cancer OR tumor OR neoplasm OR malignoma OR oncolog*) AND (posttraumatic growth OR post-traumatic growth OR post traumatic growth OR ptg OR stress-related growth*) AND (trait OR characteristics OR qualities or personality OR coping OR coping strategies OR coping skills OR coping behavior OR cope).

We decided to search for the terms posttraumatic growth and stress-related growth as these describe similar concepts. Other terms such as benefit finding, thriving, and positive adjustment also describe positive changes after negative events, but they are not defined as resulting from the struggles with the event, which is crucial to the definition of posttraumatic growth [7].

### 2.2. Eligibility Criteria

The research question and eligibility criteria were based on the PICO strategy [33]. We included studies that had (P) patients with any type of cancer and survivors who were adults at the time of cancer diagnosis as the samples. (I) Interventions were not necessary, and there was no exclusion criterion. (C) A control group was not necessary, and there was no exclusion criterion. (O) The outcomes were the relationship between PTG and any coping strategy or any personality trait.

Studies were excluded if any of the following criteria were met:

- The sample did not consist of patients with cancer and/or survivors. The sample or part of the sample was younger than 18 years at the time of cancer diagnosis.
- The study did not measure the relationship between PTG assessed via the Posttraumatic Growth Inventory (PTGI) and coping strategies and/or personality traits.
- The article was not written in English.
- The article was not a quantitative study (qualitative study, book chapters, systematic reviews, narrative reviews etc.)

### 2.3. Data Extraction

After removal of the duplicates, screening of articles followed a stepwise strategy. Two authors (K.K. and A.B.) individually screened the search results by titles and abstracts. Disagreement between the reviewers was resolved by a third author (J.G.). One author (K.K.) screened the remaining articles for the full text. Articles with any exclusion criterion were excluded after each step. The studies that were considered eligible were included in the review, and the relevant data were collected via a standardized data extraction form. It comprised the following:

- Reference (authors, publication year, country)
- Characteristics of the study population (sample size, mean age, cancer type, time since diagnosis)
- Study data (design, assessed constructs, used questionnaires)
- Results (direction of the relationship between the total PTGI score and any coping or trait variable).

This review aimed for a descriptive data analysis. After the data extraction, the methods that the studies used were tabulated and categorized to provide an overview. Afterwards, the data concerning associations between coping or traits and PTG were tabulated and categorized. We aimed to cluster coping strategies according to the second-order factors calculated in each study.

### 2.4. Quality Assessment

The quality of the included studies was assessed via the Joanna Briggs Institute (JBI) critical appraisal checklist for analytical cross-sectional studies [34]. We used the checklist for all studies (longitudinal and cross-sectional), as the longitudinal studies mostly did not assess the change in the relationships over time. Two authors (K.K. and J.G.) completed the assessment and discussed differences until consensus was reached.

## 3. Results

**Figure 1:**
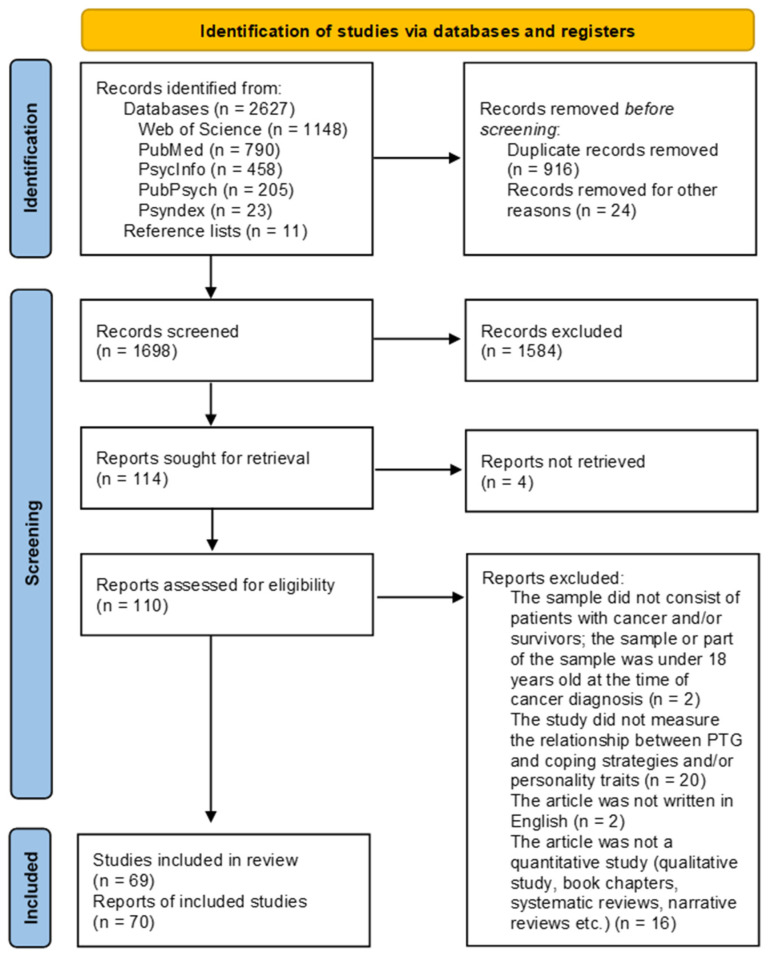
PRISMA flow diagram [32].

The literature search of the five scientific databases resulted in a total number of 2627 studies. An additional 11 studies were found in reference lists of other studies. After the removal of duplicate records, 1698 articles were left for screening. After title and abstract screening, 114 articles were left for full-text screening. Three full texts could not be retrieved. Among the full texts, 69 studies in 70 reports fulfilled the inclusion criteria and were included in the review. Two reports referred to the same study (study number 1). Figure 1 shows the study selection process in the PRISMA flow diagram [32]. Overall, the studies had good quality with some exceptions. Many studies did not describe the sample characteristics in detail. For example, they did not report the mean time since diagnosis or the mean age of the participants. Confounding factors were often not identified. One reason for this is that most studies’ main objective was not to assess the relationships we were interested in. The detailed assessment can be found in the supplementary material (Tables S1 and S2).

### 3.1. Methods Used

Articles were published between 2003 and 2022. Most articles had a cross-sectional design (70 %, Table 1). Cancer type, time since diagnosis, and number of participants varied widely across the studies. A high percentage of studies had a sample of patients with breast cancer (38%), followed by a mixed sample (31%), gynecological cancer (7%), hematologic cancer or received a hematopoietic stem cell transplant (4% each), brain cancer, craniofacial cancer, prostate cancer (3% each), colorectal cancer, head and neck cancer, hepatobiliary cancer, lung cancer, melanoma, or cancer of the oral cavity (1% each). The mean age of the sample was mainly 50 or 60 years. Some studies did not report the mean age and standard deviation. For these studies, we extracted the age range or median. Time since diagnosis ranged from a maximum of one month to a mean of over 10 years, while 49 articles did not provide information on the average time since diagnosis. The *n* ranged from 25 to 1221. Twenty-six percent of the studies were conducted in the USA, thirteen percent in China, six percent in Australia, six percent in Turkey, and the rest in other countries.

Most studies did not discriminate between genders. The studies that assessed gender differences revealed the following: While most studies (study number 6, 24, 28, 31, 37, 40, 43, 59, 62, 69, Table 1) did not reveal a significant relationship between gender and PTG, two of the included studies (study number 65, 68, Table 1) showed women to have experienced more PTG than men. The same applied to disease characteristics: while most studies (study number 24, 49, 59, 69, Table 1) did not find a relationship between PTG and disease characteristics such as time since diagnosis, type of cancer, and treatment, one study (number 27, Table 1) found higher PTG scores in breast cancer survivors than in prostate cancer survivors. However, none of the included studies assessed whether the relationship between PTG and personality/coping differed between genders and disease characteristics.

All studies used validated self-reported questionnaires. For the assessment of personality factors, 12 different tools were used assessing eight different constructs, namely, dispositional optimism, dispositional hope, dispositional gratitude, dispositional mindfulness, dispositional resilience/hardiness, positive affectivity, trait anxiety, and the Big Five (Table 2). The most reported construct was optimism, assessed by the Life Orientation Test-Revised (LOT-R) in eleven studies [104], followed by hope measured by the Hope Scale. Four studies were identified that measured the Big Five personality traits [105] (Table 2).

For the assessment of coping strategies, 20 different tools were used (Table 3). Some studies reported a variety of general coping strategies. Other studies reported cancer-specific coping strategies, emotion regulation strategies, cognitive strategies, religious coping or reflective rumination. The most frequently used tools were the Brief COPE and COPE inventory [105], followed by the Ways of Coping Inventory, the Mini Mental Adjustment to Cancer scale, and the Event-related Rumination Inventory.

### 3.2. Relationship of Variables with PTG

#### 3.2.1. Relationship of Posttraumatic Growth and Personality Traits

Looking at longitudinal as well as cross-sectional studies, there were as many positive (19) as non-significant (19) relationships between PTG and different personality traits reported (Table 4). Regarding single assessed constructs, dispositional gratitude, trait resilience/hardiness, and positive affectivity seemed to be positively related to PTG. For dispositional optimism and hope, the results were mixed, but there were slightly more studies that showed a positive relationship. For trait anxiety, no significant relationship was found. Concerning the Big Five, most studies showed non-significant correlations. For extraversion and agreeableness, no associations were found. Regarding conscientiousness and openness, two studies and one study, respectively, found negative relationships(s). For neuroticism, two studies found a negative relationship. In the four longitudinal studies (Study no. 3, 44, 60, and 62), no associations between optimism and hope measured at baseline and PTGI measured at follow-up were found [37,77,93,95]. Optimism was related to the single dimension of “personal strength” after six years [95].

#### 3.2.2. Relationship of Posttraumatic Growth and Coping Strategies

Concerning coping strategies, it was difficult to assimilate the evidence as studies were very heterogenic. In total, 57 studies investigated the relationship between PTG and coping strategies. A high variety of different assessment tools was used. To facilitate the interpretation, the coping strategies were clustered into six categories. Of the 23 studies using the Brief COPE or COPE inventory, most calculated second-order-factors based on their sample. This is recommended by the developer of the scale, Carver [23], but made it impossible to compare the studies or synthesize the results. Nine studies reported relationships between total PTGI and separate Brief COPE dimensions (Table 5). For religious coping and positive reframing/reappraisal, only positive relationships were reported. For behavioral disengagement, self-blame, and venting, no significant relationship was found in any study. Two studies found a negative relationship for denial and substance use. Interestingly, the avoidant coping strategy of self-distraction was positively related to PTG in two studies and unrelated in three studies.

The studies that calculated second-order-factors of the (Brief) COPE all reported slightly different factors (Table 6). As in previously described studies, social support seeking was mostly positively associated with PTG. Approach-oriented coping strategies were mostly positively related, whereas passive coping strategies were not related to PTG. However, there was a wide variety in which dimensions were subsumed into different second-order factors. For example, self-distraction was subsumed into the second-order factor “positive coping” in one study [41] and into “emotional avoidance strategies” in another study [42]. One study that explicitly measured coping dispositionally (general coping style) and situationally (used coping strategies in the situation) found no significant relationship between dispositional coping and PTG [43].

Table 7 summarizes the associations between PTG and diverse coping strategies that were not assessed by the Brief COPE or COPE inventories. Studies that used assessment tools other than the Brief COPE or COPE inventory to measure different general coping strategies found positive associations between PTG and all different kinds of coping strategies. The Ways of Coping Inventory [106] measures coping as a process and consisted of 66 items in the original version. Some studies used translations of this questionnaire with more or fewer items than the original. All studies calculated their own factors via factor analysis, which makes it difficult to summarize the results. All factors except one were positively related to PTG.

The Coping Responses Inventory and the Proactive Coping Inventory were used in only one and two studies, respectively. For the several calculated second-order factors, mostly positive associations with PTG were found. The only study using the Simple self-coping style questionnaire found a positive relationship of positive coping strategies and a negative relationship of negative coping strategies with PTG. The scales measuring cancer-specific coping created a more diverse picture: some negative coping strategies were negatively related to PTG whereas others were unrelated to PTG.

The emotion regulation strategy expressive suppression or inhibition was unrelated in two studies, and emotional processing was positively related to PTG in the two studies that investigated it. The results for emotional expression were unclear: one study found a positive and another one a negative relationship.

The results regarding cognitive processing were heterogenic as well. Many religious coping strategies seemed to be related to PTG, while some were not or were negatively related to PTG. Regarding reflective or deliberate rumination, 10 studies found positive relationships with PTG, and only three studies found non-significant relationships. The one study investigating dispositional reflection found positive relationships with two measures. In conclusion, seeking social support was positively related to PTG in all different self-report questionnaires except for seeking spiritual support.

Interesting findings of single longitudinal studies were the following: concerning the change of relationships over time, substance use was associated with a decrease in PTG 24 months after the diagnosis [35]. Using religion at the time of chemotherapy was related to PTG after two years [36]. Positive coping strategies were related to PTG after six months and two years, but not to PTG after seven years [41]. In some of the studies, single coping strategies were not related to PTG at follow-up [36,41]. Two studies found that different coping strategies were linked to different trajectories of PTG [43,94]. Some studies only found associations with single dimensions of PTG. For example, cognitive avoidance predicted the dimension “personal strength” after treatment completion [67]. Current deliberate rumination was found to be a mediator between coping at the time of the diagnosis and two dimensions of PTG [90]. One study found that, while the total approach coping score prior to bone marrow transplantation was not related to PTG after bone marrow transplantation, the total avoidance coping was positively related to PTG [76]. Finally, one study found that through strategies of social support seeking and using cognitive strategies, an increase in PTG in two dimensions led to a better quality of life and less depression six months after treatment completion [56].

## 4. Discussion

The aim of this review was to provide an overview of studies that assessed the relationship between coping strategies or personality traits and posttraumatic growth in patients with cancer or survivors. We first summarized the methodology of eligible studies and then categorized the reported relationships. We found a high number of studies that investigated the relationship between PTG and coping strategies and a smaller number of studies that investigated the relationship between PTG and personality factors.

Only a few personality traits have been investigated in terms of their relationship with PTG in patients with cancer or survivors to date. Regarding the Big Five personality factors, a limited number of studies investigated their relationship with PTG. Interestingly, in our synthesis, the Big Five personality traits seem unrelated to PTG. For conscientiousness, two studies found positive relationships. For openness to experience and extraversion, mostly non-significant relationships were found. Resilience, hardiness, dispositional positive affectivity, and dispositional gratitude might be key factors in the development of PTG. Optimism and hope are the two most researched variables in this context, but the results do not clearly show a positive relationship. Similar controversial results were found in a previous review in cancer patients [107]. This contradicts the findings of the original work of Tedeschi and Calhoun [5,6], who found extraversion, openness to experience, and optimism to be related. Optimism was found to have effects on PTG in a meta-analysis of mixed samples [25]. This raises the question of whether this is a unique experience in patients with cancer and survivors. Some authors describe the development of PTG itself as positive personality change [108]. On the other hand, cumulative adversity can affect personality traits such as agreeableness [109]. These two-way effects have not been investigated in any of the accumulated longitudinal studies. Personality factors such as optimism might also lead to positive psychosocial behavior change such as seeking social support [110]. In the context of psycho-oncological interventions, it could be helpful to identify individuals that are less likely to experience PTG due to their personality and support them to use positive coping strategies to make positive changes.

Most studies investigating coping strategies examined the associations cross-sectionally. The Brief COPE and COPE questionnaires were mostly used for this purpose. Most studies did not address the difference between situational and dispositional coping and only assessed situational coping. Where possible, we summarized results from all studies regarding one specific coping strategy. We found that promoting the use or seeking of social support, religious coping, and positive reframing and reflection during illness could contribute to the development of PTG. Comparing different ways of assessing coping, seeking social support was nearly consistently positively related to PTG. Moreover, religious coping and social support seeking were found to have effects on PTG in a meta-analysis of mixed samples [25]. This is also in line with the systematic reviews of Kolokotroni [28] and Rajandram [27], who conducted their studies on patients with breast cancer and oral cavity cancer, respectively. They found seeking of social support, religious coping, and reframing to be related to PTG. In contrast to these studies, we found acceptance to be rather unrelated, with mixed results for humor and active coping. We also found that individuals who used the strategies reappraisal of emotions and reflection overall, seemed to report more PTG. Interestingly, strategies that seem negative, such as denial, distraction, avoidance, suppression, self-blame, and substance use, were not related to PTG when measured via the COPE inventory or Brief COPE inventory. These often so-called “dysfunctional” or “negative” coping strategies might not be so dysfunctional when it comes to positive changes following adversity. In total, only a few studies found negative associations of PTG with any coping strategy. In summary, a practical application of these findings would be to promote seeking social support in interventions and to reduce barriers in this regard.

There are a few limitations in our review. The heterogeneous methodology of the studies in the assessment of a relationship between coping strategies and PTG, such as questionnaires and the calculation of second-order factors, made it difficult to summarize all results and to compare different samples. In particular, the data extraction concerning associations between PTG and coping strategies posed a challenge. The heterogeneous results mirror the heterogeneous methods. The strategy of conducting factor analysis within one’s own sample instead of using a given structure of a questionnaire has been endorsed by researchers who developed and validated the scales [23,111]. PTG, as well as coping strategies and personality, develops over time in response to situations [112,113,114]. Measuring these factors at one time point is thus not sufficient to explore their dynamic changes. Due to the quantity of the studies, we could not discuss the inclusion of additional confounding factors in the studies.

Future studies should report the correlations for individual dimensions in addition to the second-order factors so that a comparison with other samples is possible. Further longitudinal studies are necessary to capture the change in PTG and its influence on relationships with other variables over time. Therefore, it is necessary to assess all variables at all time points to observe the trajectories of PTG, coping strategies, and personality traits. As found in single included studies, longitudinal studies should further explore relationships with single PTG dimensions compared to the total PTGI score and the difference of relationships with personality traits or coping strategies across different types of PTG trajectories. Confounding factors should be considered and included in analyses. Few studies addressed the difference between dispositional coping and situational coping. It could be of use to differentiate between those concepts in future research. To answer the question, whether certain personality traits do not play a role in PTG in patients with cancer, further studies concentrating on these variables are necessary. It is important to note that the concept of PTG as operationalized by Tedeschi and Calhoun does not assess certain special dimensions that exist in serious illness, such as positive health behavior changes. A concentration on this original concept could thus lead to ignore other important aspects of change that are unique in the experience of cancer [110]. While we focused on quantitative studies that used the validated and often cited PTGI in our review, qualitative studies exploring other dimensions specific to cancer might complete this field of research. Another possible benefit of qualitative studies could be the assessment of coping strategies that are perceived as useful by patients with cancer and survivors themselves for positive adjustment.

This review supplements the growing body of evidence on the topic of PTG in patients with cancer and survivors. This field of research is rapidly evolving. Our results could be of use for the development of psycho-oncological interventions that should not only aim to reduce distress but also address the possible development of PTG. Enhancing PTG might even have an indirect effect on distress itself. As Shakespeare-Finch states: “…Positive and negative post-trauma outcomes can co-occur. A focus only on PTSD symptoms may limit or slow recovery and mask the potential for growth.” [14]. One aspect could be the enhancement of coping strategies that are diverse and variable and can be learned over time.

## Figures and Tables

**Table 1:** Sample and study characteristics.

No.	First Author (Year)	*n*	Country	Type of Cancer	Mean Time since Diagnosis (SD) ^2^	Mean Age in Years (SD) ^2^	Study Design
1	Bourdon (2019) ^1^ [35]	78	France	Melanoma	Max. 1 month	51 (NR)	L
1	Bourdon (2019) ^1^ [35]	215	France	Breast	Max. 1 month	53 (NR)	L
2	Bussell (2010) [36]	59	USA	Breast	NR, currently in chemotherapy	50, range: 28–76	L
3	Moore (2011) [37]	202	USA	Hepatobiliary	At diagnosis	63 (NR)	L
4	Wilson (2014) [38]	514	Australia	Prostate	7.5 years (4.66)	70 (NR)	C
5	Tallman (2013) [39]	98	USA	Mixed	325 days (564)	Range: 18–80	L
6	Li (2019) [40]	330	China	Brain	NR	40 (NR)	C
7	Hamama-Raz (2019) [41]	198	Israel	Breast	NR	52 (10.9)	L
8	Kim (2021) [42]	114	Korea	Brain	NR	55 (11.5)	C
9	Cheng (2020) [43]	84	Taiwan	Breast	NR	50 (8.7)	L
10	Leong Abdullah (2019) [44]	195	Malaysia	Mixed	NR	53 (10.3)	C
11	Yu (2014) [45]	230	China	Mixed	NR	64 (3.1)	C
12	Zhang (2021) [46]	532	China	Lung	NR	Median = 57	C
13	Koutrouli (2016) [47]	202	Greece	Breast	NR	61 (11.3)	C
14	Oh (2021) [48]	148	Korea	Gynecological	NR	62 (9.4) 69 (8.6) 68 (2.2) 70 (9.9) ^4^	C
15	Wang (2016) [49]	139	China	Mixed	29.36 months (47.85)	58 (12.3)	C
16	Liu (2018) [50]	202	China	Breast	NR	48 (8.9)	C
17	Lelorain (2010) [51]	307	France	Breast	10 years (2.8)	62 (7.9)	C
18	Büyükaşik-Çolak (2012) [52]	90	Turkey	Breast	12.54 months (NR)	45 (8.7)	C
19	Bellur (2018) [53]	134	Turkey	Breast	NR	45 (8.2)	C
20	Lianchao (2020) [54]	309	China	Mixed	NR	59 (3.3)	C
21	Cao (2018) [55]	201	China	Mixed	NR	50 (11.2)	C
22	Silva (2012) [56]	50	Portugal	Breast	NR	52 (8.3)	L
23	Gori (2021) [57]	154	Italy	Mixed	NR	51 (11.3)	C
24	Baník (2014) [58]	109	Slovakia	Hematologic	NR	48 (14.6)	C
25	Tu (2019) [59]	201	Taiwan	Breast	39.14 months (18.45)	52 (9.7)	C
26	Manne (2004) [60]	162	USA	Breast	NR	49 (NR)	L
27	Caspari (2017) [61]	169	USA	Mixed	NR	61 (11.4)	C
28	Cormio (2017) [13]	540	Italy	Mixed	NR	57 (11.0)	C
29	Zhou (2021) [62]	344	China	Gynecological	NR	Range: 21–78 years	C
30	Villanova Quiroga (2020) [63]	84	Brazil	Breast	Median = 4 years (percentiles 25–75: 2–10)	55 (12.7)	C
31	Salsman (2009) [64]	55	USA	Colorectal	1.07 years (0.19)	66 (12.7)	L
32	Zhang (2020) [65]	1221	China	Mixed	8.36 years (4.67)	62 (8.6)	C
33	Baglama (2010) [66]	31	North Cyprus	Breast	Diagnosis within the past 5 years	51 (11.6)	C
34	Carboon (2005) [67]	62	Australia	Hematologic	NR	43 (14.3)	L
35	Cohen (2011) [68]	124	Israel	Breast	NR	70 (17.4)	C
36	Boyle (2017) [69]	175	USA	Breast	18.61 months (2.88)	53 (8.0)	C
37	Ho (2004) [70]	188	Hong Kong	Mixed	NR	49 (0.6)	C
38	Smith (2008) [71]	183	USA	Gynecological	10.30 years (5.01)	51 (9.1)	C
39	Thornton (2006) [72]	82	USA	Prostate	NR	61 (7.4)	L
40	Baghjari (2017) [73]	120	Iran	Mixed	NR	47 (14.7)	C
41	Bellizzi (2006) [74]	224	USA	Breast	NR	60 (12.0)	C
42	MoshirPanahi (2020) [75]	300	Iran	Mixed	Range: 5–84 months post-diagnosis	53 (27.6)	C
43	Widows (2005) [76]	72	USA	Receiving hematopoietic stem cell transplant	24.05 months (10.01) post-BMT at time of follow-up	48 (10.0)	L
44	Danhauer (2013) [77]	653	USA	Breast	Median = 4.7 months, range = 0.1–7.3	55 (12.6)	L
45	Strack (2010) [78]	128	Germany	Mixed	NR	55 (12)	C
46	Roohi (2020) [79]	265	Iran	Mixed	NR	NR	C
47	Morris (2011) [80]	313	Australia	Mixed	2.92 years (1.86), range: 1.5–4	62 (12.1)	C
48	Tomita (2017) [81]	157	Japan	Breast	64.13 (45.4) months	59 (10.1)	C
49	Scrignaro (2011) [82]	41	Italy	Mixed	NR	52 (7.7)	L
50	Fujimoto (2021) [83]	80	Japan	Breast	NR, range: 2–10 years	NR	C
51	Bozo (2009) [84]	104	Turkey	Breast	29.15 months (49.88)	46 (9.2)	C
52	Ogińska-Bulik (2017) ^3^ [85]	60	Poland	Craniofacial	NR	50 (17.7)	C
53	Gall (2011) [86]	93	Canada	Breast	NR	61 (11.3)	L
54	Schmidt (2012) [87]	54	USA	Mixed	4.5 (2.8) years	53 (10.5)	C
55	Schroevers (2008) [88]	113	Malaysia	Mixed	45 months (40.53)	52 (11.1)	C
56	Aflakseir (2016) [89]	120	Iran	Breast	4.6 years	51 (10.0)	C
57	Ogińska-Bulik (2019) [90]	71	Poland	Mixed	NR	49 (12.7)	L
58	Ogińska-Bulik (2018) ^3^ [91]	60	Poland	Craniofacial	NR	50 (17.7)	C
59	Ho (2011) [92]	50	Hong Kong	Oral cavity	3.6 years (0.34)	60 (13.1)	C
60	Sears (2003) [93]	92	USA	Breast	28.47 weeks (13.38)	52 (10.3)	L
61	Danhauer (2015) [94]	653	USA	Breast	NR	54, range: 25–96	L
62	Tallman (2010) [95]	25	USA	Receiving hematopoietic stem cell transplant	NR	37 (10.3) at time of transplantation	L
63	Hill (2017) [96]	59	USA	Gynecological	58.90 months (56.95)	50 (10.6), range: 28–74	C
64	Morris (2007) [97]	335	Australia	Mixed	Range: 1.5–4 years	63 (12.2)	C
65	Jaarsma (2006) [98]	294	Netherlands	Mixed	3.90 years (2.50)	56 (12.2), range: 21–84	C
66	Ruini (2013) [99]	67	Italy	Breast	7 years (4.4)	57 (11.7)	C
67	Schwartz (2022) [100]	430	USA	Receiving hematopoietic stem cell transplant	NR	53, range: 19–74	L
68	Nik Jaafar (2021) [101]	200	Malaysia	Head and neck	NR	NR	L
69	Boyacıoğlu (2022) [102]	111	Turkey	Hematologic	NR	50 (16.0)	C
70	Karimzadeh (2021) [103]	210	Iran	Breast	NR	48 (10.5), range: 41–50	C

NR = not reported. C = cross-sectional. L = longitudinal. ^1^ Two separate subsamples in this study. ^2^ Some studies did not report the mean and/or standard deviation. For these studies, the age range or median is given if available. ^3^ Two reports of the same study. ^4^ No total mean or SD reported. Mean and SD reported for each of the four analyzed subgroups.

**Table 2:** Assessment tools for personality (self-report questionnaires).

Construct	Assessment Tool	Used in Study ^1^	Total Number of Studies
Dispositional optimism	Life Orientation Test-Revised (LOT-R)	3, 10, 18, 24, 38, 41, 44, 51, 59, 60, 62	11
Dispositional hope	Hope Scale (HS)	10, 33, 41, 59, 60	5
	Adult Hope Trait Scale (AHTS)	24	1
Dispositional gratitude	The Gratitude Questionnaire—Six Item Form (GQ-6)	45, 66	2
Dispositional mindfulness	Mindfulness Attention Awareness Scale (MAAS)	16, 20	2
Trait resilience/hardiness	Connor-Davidson Resilience Scale (CD-RISC-10)	23, 25	2
	Ahvaz psychological hardiness scale	56	1
Positive affectivity	Positive and Negative Affect Schedule (PANAS)	17	1
Trait anxiety	State-Trait Anxiety Inventory (STAI-Y)	28	1
Big Five	NEO Five-Factor Inventory (NEO-FFI)	52, 58, 65	3
	Big Five Inventory (BFI)	45	1
	Ten-Item Personality Inventory (TIPI)	23	1

^1^ study numbers: see Table 1.

**Table 3:** Assessment tools for coping strategies (self-reported questionnaires).

Construct(s)	Assessment Tool	Used in Study ^1^	Total Number of Studies
Different general coping strategies	Brief COPE	1, 2, 6, 17, 21, 22, 36, 39, 41, 44, 46, 49, 54, 55, 61, 67, 68	17
	COPE inventory	5, 23, 26, 47, 60, 64	6
	Ways of Coping Inventory (WCI)	18, 19, 30, 48	4
	Coping responses inventory (CRI)	40, 43	2
	Simple self-coping style (SCSQ)	32	1
	Proactive Coping Inventory	50	1
Cancer-specific coping	Mini Mental Adjustment to Cancer scale	9, 25, 34, 37	4
	Cancer Coping Questionnaire (CCQ)	8, 14	2
	Medical Coping Modes Questionnaire (MCMQ)	29	1
Emotion regulation	Emotion Regulation Questionnaire	12, 45, 70	3
	Emotional expression and processing scale	26, 35	2
	Emotion Regulation scale	11	1
Cognitive strategies	Cognitive Processing of trauma scale (CPOTS)	27, 42	2
	Cognitive processing scale	35	1
	Meaning-Focused Coping Questionnaire (MFCQ)	15	1
	Cognitive Emotion Regulation Questionnaire (CERQ)	7	1
Religious coping	RCOPE	53, 69	2
Reflective rumination	Event-Related Rumination Inventory (ERRI)	4, 52, 57, 63	4
	Rumination-Reflection-Questionnaire (RRQ)	30, 58	2
	State Level Measure of Reflection and Brooding	13	1
	Rumination scale	31	1
	Rumination inventory	47	1

^1^ study numbers: see Table 1.

**Table 4:** Relationships of different personality traits with the PTGI total score.

	Direction of Found Relationship: Study Number ^1^		
Construct	Positive	Negative	n.s.
Dispositional optimism	3, 10, 18, 24, 51, 59		38, 41, 44, 60, 62
Dispositional hope	10, 33, 59		41, 60
Dispositional gratitude	45, 66		
Dispositional mindfulness	20	16	
Trait resilience/hardiness	23, 25, 56		
Positive affectivity	17		
Trait anxiety			28
Big Five: Openness	65		23, 52, 58
Big Five: Conscientiousness	23, 58		52
Big Five: Extraversion			23, 52, 58
Big Five: Agreeableness			23, 52, 58
Big Five: Neuroticism		23, 58	52, 65

n.s. = not significant. ^1^ study numbers: see Table 1.

**Table 5:** Relationships of single (Brief) COPE dimensions with the PTGI total score.

	Direction of Relationship: Study Number ^1^		
(Brief) COPE Dimension	Positive	Negative	n.s.
Behavioral disengagement			2, 54, 55, 67
Self-blame			2, 17, 49, 54, 55
Denial	17	68	2, 49, 55, 67
Use of instrumental support	2, 49, 55		39, 54
Use of emotional support	2, 39, 49, 55		54
Venting			2, 49, 54, 55
Religion	2, 17, 54, 55		
Active coping	54, 55, 67		2, 39
Planning	49, 55, 67, 68		2, 54
Self-distraction	49, 67		2, 54, 55
Positive reframing/reappraisal	2, 26, 39, 55, 67		
Humor	49, 55		2, 54
Acceptance	67, 68		2, 39, 49, 54, 55
Substance use		1	2, 17, 54, 67

n.s. = not significant. ^1^ study numbers: see Table 1.

**Table 6:** Relationships of (Brief) COPE second-order factors with the PTGI total score.

Second-Order Factor	Contained (Brief) COPE Dimensions	Direction of Relationship: Study Number ^1^		
		Positive	Negative	n.s.
Positive attitude	NR	23		
Positive	Humor, positive reframing, acceptance	17		
Positive coping	Active coping, planning, self-distraction, positive reframing, humor, acceptance	1		
Dispositional meaning-making coping	e.g., positive reinterpretation			5
Situational meaning-making coping	e.g., positive reinterpretation	5		
Cognitive coping	Acceptance, humor, planning and positive reframing			22
Problem-focused coping	NR	46		
Active coping	Use of emotional support, positive reframing, active coping, planning, acceptance	6		
Active coping	Active coping, self-distraction, planning	17		
Active adaptive coping	Self-distraction, active coping, seeking emotional and instrumental support, venting, positive reframing, planning, acceptance, and turning to religion	41		
Active-adaptive coping	Self-distraction, active coping, emotional support, instrumental support, venting, positive reframing, planning, turning to religion	44, 61		
Situational active/adaptive coping	e.g., planning coping	5		
Dispositional active/adaptive coping	e.g., planning coping			5
Adaptive coping	Active coping, planning, positive reframing	21 (except spiritual change)		
Approach-oriented coping	Active coping, planning, acceptance, instrumental and emotional social support seeking	36		
Approach coping	NR	23		
Emotional engagement strategies	Active coping, positive reframing, emotional processing, acceptance, planning	67		
Emotion-focused coping	NR	46		
Emotional coping	Using instrumental support, using emotional support, venting, religion	1		
Relational	Emotional support, instrumental support, venting	17		
Social Support seeking	Seeking emotional support, seeking instrumental support	47		22
Social Support	NR	23		
Social support	emotional social support seeking, instrumental social support seeking	64		
Turning to religion	NR			23
Avoidant coping	Denial, alcohol/drug use, behavioral disengagement, venting	6		
Avoidance strategies	NR			23
Avoidance coping	NR	46		
Emotional avoidance strategies	Distraction, denial, behavioral disengagement, substance use			67
Passive coping	Self-blame, denial, behavioral disengagement			44, 61
Negative coping	Behavioral disengagement, self-blame, denial			1
Maladaptive coping	Denial, alcohol/drug use, behavioral disengagement			41
Situational maladaptive coping	e.g., denial coping			5
Dispositional maladaptive coping	e.g., denial coping			5

n.s. = not significant. NR = not reported. ^1^ study numbers: see Table 1.

**Table 7:** Relationships of further coping strategies with the PTGI total score.

Assessment Tool	Second-Order-Factor	Direction of Relationship: Study Number ^1^		
		Positive	Negative	n.s.
Different General Coping Strategies				
Ways of Coping Inventory (WCI)	Problem-focused coping	18		
	Emotion-focused coping	18		
	Problem focused/optimistic coping style	19		
	Fatalistic coping	19		
	Helplessness coping			19
	Comfort	30		
	Seclusion	30		
	Self-control	30		
	Social support	30		
	Responsibility acceptance	30		
	Dodge–escape	30		
	Problems resolution	30		
	Positive reevaluation	30		
	Self-restraining coping	48		
	Distancing coping	48		
	Positive coping	48		
	Coping by depending on others	48		
Coping Responses Inventory (CRI)	Cognitive assessment focused coping	40		
	Social support seeking coping	40		
	Problem-solving coping	40, 43		
	Emotional inhibition coping			40
	Somatic inhibition coping			40
	Approach coping	43		
	Avoidance coping	43		
Proactive Coping Inventory	Proactive coping	50		
	Reflective coping	50		
	Strategic planning	50		
	Preventive coping	50		
	Instrumental support seeking	50		
	Emotional support seeking	50		
	Avoidance coping	50		
Simple self-coping style (SCSQ)	Positive coping style	32		
	Negative coping style		32	
Cancer-specific coping				
Mini Mental Adjustment to Cancer Scale (Mini-MAC)	Negative emotion coping		25	37
	Positive attitude coping	37		
	Cognitive avoidance			9, 25, 34, 37
	Hopelessness–helplessness		9	
	Fatalism	9		
	Anxious preoccupation			9
	Fighting spirit			9
	Positive-Acceptance coping	25		
Cancer Coping Questionnaire	Individual coping	8, 14		
	Interpersonal coping	8, 14		
Medical Coping Modes Questionnaire (MCMQ)	Confrontation coping	12, 29		
	Avoidance coping	12, 29		
	Acceptance-resignation		12, 29	
Emotion regulation				
Emotion Regulation Scale	Expressive revealing	11		
	Expressive suppression			11
Emotion Regulation Questionnaire (ERQ)	Cognitive reappraisal of emotion	12, 45		
	Expression inhibition			12
Emotional Approach Coping Scales (EAC)	Emotional expression		36	
	Emotional processing	36		
Emotional expression and processing scale	Emotional expression	35		
	Emotional processing	35		
Cognitive strategies				
Cognitive Processing of trauma scale (CPOTS)	Positive cognitive processing	27, 42		
	Negative cognitive processing		27	42
Cognitive processing scale		35		
Meaning-Focused Coping Questionnaire (MFCQ)		15		
Cognitive Emotion Regulation Questionnaire (CERQ)	Positive coping strategies			7
	Negative coping strategies			7
Religious coping				
RCOPE	Benevolent religious reappraisal (meaning)	53		
	Collaborative Religious Coping (Control)			53
	Active Surrender (Control)		53	
	Passive Religious Deferral (Control)		53	
	Pleading for Direct Intercession (Control)	53		
	Seeking Spiritual Support (Comfort)			53
	Religious Focus (Comfort)	53		
	Spiritual Discontent (Comfort)	53		
	Religious Helping (Intimacy)			53
	Seeking Religious Direction (Life Transformation)	53		
	Negative religious coping	69		
Rumination				
Rumination Scale	Cognitive rehearsal	31		
Rumination-Reflection Questionnaire (RRQ)	Dispositional reflection	58		
	Deliberate/reflective rumination	20		58
Event Related Rumination Inventory (ERRI)	Dispositional reflection	58		
	Deliberate/reflective rumination	4, 20, 58, 63		52, 57
Rumination Inventory (RI)	Deliberate rumination on benefits	47		
State Level Measure of Reflection and Brooding	Reflective rumination	13		

n.s. = not significant. ^1^ study numbers: see Table 1.

## Supplementary Materials

The following supporting information can be downloaded at: https://www.mdpi.com/article/10.3390/curroncol29120754/s1, Table S1: Quality assessment via the JBI critical appraisal checklist for analytical cross-sectional studies. Table S2: Data extraction.
