# Supplementary material for: Personality Traits and Coping Strategies Relevant to Posttraumatic Growth in Patients with Cancer and Survivors: A Systematic Literature Review
Source: Curr Oncol. 2022 Dec 6;29(12):9593–612. doi: 10.3390/curroncol29120754 (PMC9776882; doi:10.3390/curroncol29120754)
Supplement: Supplementary file 1 [file curroncol-29-00754-s001.zip › Table S1_supplementary material.pdf]

Table S1. Quality assessment via the JBI critical appraisal checklist for analytical cross sectional studies

Study no.	First author (year)	Were the criteria for inclusion in the sample clearly defined?	Were the study subjects and the setting described in detail?	Was the exposure measured in a valid and reliable way?	Were objective, standard criteria used for measurement of the condition?	Were confounding factors identified?	Were strategies to deal with confounding factors stated?	Were the outcomes measured in a valid and reliable way?	Was appropriate statistical analysis used?
1	Bourdon (2019)	Y	Y	Y	Y	N	N.a.	Y	Y
2	Bussell (2010)	Y	Y	Y	Y	N	N.a.	Y	Y
3	Moore (2011)	Y	Y	Y	Y	N	N.a.	Y	Y
4	Wilson (2014)	Y	Y	Y	Y	Y	Y	Y	Y
5	Tallman (2013)	Y	N	Y	Y	N	N.a.	Y	Y
6	Li (2019)	Y	N	Y	Y	Y	Y	Y	Y
7	Hamama-Raz (2019)	Y	N	Y	Y	N	N.a.	Y	Y
8	Kim (2021)	Y	N	Y	Y	Y	U	Y	Y
9	Cheng (2020)	Y	N	Y	Y	Y	Y	Y	Y
10	Leong Abdullah (2019)	Y	N	Y	Y	Y	N	Y	Y
11	Yu (2014)	Y	N	Y	Y	Y	Y	Y	Y
12	Zhang (2021)	Y	Y	Y	Y	N	Y	Y	Y
13	Koutrouli (2016)	Y	N	Y	Y	Y	Y	Y	Y
14	Oh (2021)	Y	Y	Y	Y	Y	Y	Y	Y
15	Wang (2016)	Y	Y	Y	Y	Y	N	Y	Y
16	Liu (2018)	Y	N	Y	Y	N	N.a.	Y	Y
17	Lelorain (2010)	Y	Y	Y	Y	Y	Y	Y	Y
18	Büyükaşik-Çolak (2012)	Y	Y	Y	Y	N	N.a.	Y	Y
19	Bellur (2018)	Y	Y	Y	Y	N	N.a.	Y	Y
20	Lianchao (2020)	Y	N	Y	Y	N	N.a.	Y	Y
21	Cao (2018)	Y	N	Y	Y	N	N.a.	Y	Y

Study no.	First author (year)	Were the criteria for inclusion in the sample clearly defined?	Were the study subjects and the setting described in detail?	Was the exposure measured in a valid and reliable way?	Were objective, standard criteria used for measurement of the condition?	Were confounding factors identified?	Were strategies to deal with confounding factors stated?	Were the outcomes measured in a valid and reliable way?	Was appropriate statistical analysis used?
22	Silva (2012)	Y	N	Y	Y	N	N.a.	Y	Y
23	Gori (2021)	Y	N	Y	Y	N	N.a.	Y	Y
24	Baník (2014)	Y	N	Y	Y	N	N.a.	Y	Y
25	Tu (2019)	Y	Y	Y	Y	Y	U	Y	Y
26	Manne (2004)	Y	N	Y	Y	Y	Y	Y	Y
27	Caspari (2017)	Y	N	Y	Y	Y	Y	Y	Y
28	Cormio (2017)	Y	N	Y	Y	N	N.a.	Y	Y
29	Zhou (2021)	Y	Y	Y	Y	N	N.a.	Y	Y
30	Villanova Quiroga (2020)	Y	Y	Y	Y	Y	Y	Y	Y
31	Salsman (2009)	Y	Y	Y	Y	Y	Y	Y	Y
32	Zhang (2020)	Y	Y	Y	Y	N	N.a.	Y	Y
33	Baglama (2010)	Y	Y	Y	Y	N	N.a.	Y	Y
34	Carboon (2005)	Y	N	Y	Y	Y	U	Y	Y
35	Cohen (2011)	Y	N	Y	Y	Y	Y	Y	Y
36	Boyle (2017)	Y	Y	Y	Y	Y	Y	Y	Y
37	Ho (2004)	Y	N	Y	Y	N	N.a.	Y	Y
38	Smith (2008)	Y	Y	Y	Y	Y	U	Y	Y
39	Thornton (2006)	Y	N	Y	Y	N	N.a.	Y	Y
40	Baghjari (2017)	N	N	Y	Y	N	N.a.	Y	Y
41	Bellizzi (2006)	Y	N	Y	Y	Y	Y	Y	Y
42	MoshirPanahi (2020)	Y	Y	Y	Y	N	N.a.	Y	Y
43	Widows (2005)	Y	Y	Y	Y	N	N.a.	Y	Y
44	Danhauer (2013)	Y	Y	Y	Y	Y	U	Y	Y

Study no.	First author (year)	Were the criteria for inclusion in the sample clearly defined?	Were the study subjects and the setting described in detail?	Was the exposure measured in a valid and reliable way?	Were objective, standard criteria used for measurement of the condition?	Were confounding factors identified?	Were strategies to deal with confounding factors stated?	Were the outcomes measured in a valid and reliable way?	Was appropriate statistical analysis used?
45	Strack (2010)	Y	N	Y	Y	Y	Y	Y	Y
46	Roohi (2020)	Y	N	Y	Y	N	N.a.	Y	Y
47	Morris (2011)	Y	Y	Y	Y	N	N.a.	Y	Y
48	Tomita (2017)	Y	Y	Y	Y	N	N.a.	Y	Y
49	Scrignari (2011)	Y	N	Y	Y	Y	N.a.	Y	Y
50	Fujimoto (2021)	Y	N	Y	Y	Y	Y	Y	Y
51	Bozo (2009)	Y	Y	Y	Y	N	N.a.	Y	Y
52	Ogińska-Bulik (2017)	Y	N	Y	Y	N	N.a.	Y	Y
53	Gall (2011)	Y	N	Y	Y	Y	N.a.	Y	Y
54	Schmidt (2012)	Y	Y	Y	Y	Y	Y	Y	Y
55	Schroevers (2008)	Y	Y	Y	Y	N	N.a	Y	Y
56	Aflakseir (2016)	Y	Y	Y	Y	N	N.a.	Y	Y
57	Ogińska-Bulik (2019)	Y	N	Y	Y	N	N.a.	Y	Y
58	Ogińska-Bulik (2018)	Y	N	Y	Y	N	N.a.	Y	Y
59	Ho (2011)	Y	Y	Y	Y	N	N.a.	Y	Y
60	Sears (2003)	Y	Y	Y	Y	N	N.a.	Y	Y
61	Danhauer (2015)	Y	N	Y	Y	N	N.a.	Y	Y
62	Tallman (2010)	Y	N	Y	Y	N	N.a.	Y	Y
63	Hill (2017)	Y	Y	Y	Y	N	N.a.	Y	Y
64	Morris (2007)	Y	Y	Y	Y	N	N.a.	Y	Y
65	Jaarsma (2006)	Y	Y	Y	Y	Y	U	Y	Y
66	Ruini (2013)	Y	Y	Y	Y	N	N.a.	Y	Y
67	Schwartz (2022)	Y	N	Y	Y	N	N.a.	Y	Y
68	Nik Jaafar (2021)	Y	N	Y	Y	Y	Y	Y	Y

Study no.	First author (year)	Were the criteria for inclusion in the sample clearly defined?	Were the study subjects and the setting described in detail?	Was the exposure measured in a valid and reliable way?	Were objective, standard criteria used for measurement of the condition?	Were confounding factors identified?	Were strategies to deal with confounding factors stated?	Were the outcomes measured in a valid and reliable way?	Was appropriate statistical analysis used?
69	Boyacıoğlu (2022)	Y	N	Y	Y	Y	U	Y	Y
70	Karimzadeh (2021)	Y	N	Y	Y	N	N.a.	Y	Y

** Y = Yes; N = No; U = Unclear; N.a. = Not applicable*
